# Vaccine hesitancy among mothers of under-five children in Coastal South India: a facility-based cross-sectional study

**DOI:** 10.12688/f1000research.28293.3

**Published:** 2021-09-16

**Authors:** Rekha Thapar, Nithin Kumar, Priya Surendran, Aleemath Shahdiya, Vibha Mahendran, Ranjitha Ramesh, Deepali J. Shetty, Bhaskaran Unnikrishnan, Prasanna Mithra, Ramesh Holla, Darshan Bhagwan, Avinash Kumar

**Affiliations:** 1 Department of Community Medicine, Kasturba Medical College, Mangalore, Manipal Academy of Higher Education, Manipal, India; 2 Kasturba Medical College, Mangalore, Manipal Academy of Higher Education, Manipal, India

**Keywords:** Vaccine Hesitancy, PACV, Mothers, Vaccination coverage, Mangalore

## Abstract

**Background: **Vaccine hesitancy (VH) has been identified by the World Health Organization as one among the top ten threats to global public health. There is limited literature regarding VH from developing countries like India.

**Methods: **In this facility based cross-sectional study, 172 mothers of under-five children were assessed regarding VH using the parental attitude towards childhood vaccination questionnaire (PACV).

**Results: **The prevalence of VH was 3.4% (n=6). Only 7.6% (n=13) of the study participants had ever refused vaccination for their child and the most common reason cited for their refusal was that they thought it was not safe (n=6). Government health facilities was the place of routine immunization for 60.5% (n=104) participants. Health care providers (n =79, 46%) were the major source of information regarding vaccines.

**Conclusions: **Our study highlights the presence of very low prevalence of VH in Mangalore compared to similar studies from India and other parts of the world. The limited number of participants had refused vaccination due to concerns related to vaccine safety.

## Introduction

One of the most effective and economical health interventions in recent times, immunization, has brought down the incidence of under-five morbidity and mortality. The effectiveness of a vaccine is subject to a significant number of people in the community being immunized. Unvaccinated children make those vaccinated susceptible to a disease due to waning herd immunity, as seen in poliomyelitis ( Grassly *et al.*, 2012). To achieve maximum immunization coverage, the Global Vaccine Action Plan 2011-2020 (GVAP) called for countries to achieve =90% immunization coverage by 2020 for all vaccines by incorporating it in their National Immunization Schedule ( Peck *et al.*, 2019). The highly ambitious Sustainable Development Goals (SDGs), to be achieved by 2030, includes immunization indictors to measure the progress made by countries in their journey towards ensuring healthy lives and promote well-being of all age groups. The percentage of children up to one year of age who have received three doses of diphtheria-tetanus and pertussis vaccine (DTP3) is taken as proxy indicator for full immunization ( WHO, 2017). These estimates of DTP3 are used to monitor the performance of the health system, measure the effectiveness of the immunization program in terms of service delivery, and implement strategies for disease eradication and elimination ( Measure Evaluation, n.d.). The global coverage for DTP1 and DTP3 in 2018 was estimated to be 90% and 86% and 86% for the first dose of measles-containing vaccine (MCV1). In India, even though maternal and neonatal tetanus has been eliminated and the country certified polio free, the coverage of  routine immunization continues to remain low (UNICEF, n.d.). Only 62% of children were fully immunized, with the coverage of DTP3 and measles first dose estimated at 78.4% and 81.1%, respectively (Ministry of Health and Family Welfare, [ MoHFW, 2015a]).

The lower vaccination coverage and dropouts may be attributed to populations who are either hard to reach or hard to vaccinate. Hard to reach are those residing in conflict zones or in difficult geographical terrain where vaccination is poor due to inadequate or irregular supply of vaccines. Hard to vaccinate population are those who despite having access to vaccination do not get their children immunized. This group poses a big challenge to immunization programs across the world ( Peck, 2019).

“Vaccine hesitancy [VH] refers to the delay in acceptance or refusal of vaccinations despite availability of vaccination services” ( WHO, 2014). Vaccine hesitant individuals may be those who have doubts about the vaccine but still get their children vaccinated, or individuals who either refuse or delay a vaccine but receive others, or individuals who reject all vaccines. VH is usually suspected to be present when the immunization coverage is low despite having adequate health services and provision of regular health communication by grass root level workers. This has threatened to reverse the progress made over the years in achieving adequate immunization coverage (WHO, n.d.1). VH has been identified by the WHO as one among the top ten threats to global public health (WHO, n.d.2), and was reported to be a significant problem by 74% of WHO member countries. These countries cited issues related to risk and benefits of vaccines as the most important reason for VH ( MacDonald, Butler & Dube, 2018). However, VH is a complex and context specific phenomenon driven by behavioral and social factors which influence an individual’s decision of accepting a vaccine, and not just limited to the safety concerns regarding a vaccine ( MacDonald, Butler & Dube, 2018, Thomson, Robinson & Vallee-Tourangeau, 2016). The reason for VH also varies across geographical regions and for different vaccines. The reasons for VH have been explained by the three ‘C’s model - confidence, complacency and convenience. Lack of confidence in health systems and/or professionals regarding immunization services and provision of information complacency resulting in a belief that the vaccine is no longer needed once there is a decrease in incidence of disease, and ( Edwards and Hackelle, 2016) vaccination convenience in terms of availability, affordability, accessibility and quality of services provided, and communication offered by health workers can also affect vaccination uptake ( MacDonald, 2015).

Adverse effects of the vaccines, pain and fever following immunization, wrong and misleading information from mass media, unfavorable incidents following immunization reported by relatives and friends and lack of awareness and social factors are the reasons for VH in many countries ( LaClair, Smith & Woodword, 2014).

There is limited literature regarding VH from Mangalore, where the proportion of children fully immunized (children between 12-23 months who have received measles, BCG and 3 doses of DPT and OPV) is 77.3% This is higher than the overall state average of 62.6% (Ministry of Health and Family Welfare, [ MoHFW, 2015c]) and the National average of 62.9% (Ministry of Health and Family Welfare, [ MoHFW, 2015a]). However, for a district with high literacy rate and, the immunization coverage rates are expected to be more than 80%.

With this background, we carried out this study to find the prevalence and reasons for VH among mothers of under-five children in Mangalore.

## Methods

### Study area

Mangalore, the major commercial and district headquarter of Dakshina Kannada in the South Indian state of Karnataka, India is known for its numerous educational institutes. It has a literacy rate of 93.4% with male literacy being 96.03% and female literacy 90.84%, which is better than the national urban average of 85% ( Census, 2001). The performance of the district in achieving key health care indicators including immunization coverage is also better than the state and national average.

### Participants

In this facility-based cross-sectional study, mothers of under-5 children visiting hospitals affiliated to Kasturba Medical College, Mangalore were assessed regarding VH. The study was carried out during the months of March and April 2017. Based on a previous study ( Dubé *et al*., 2016), where VH was found to be 40.2%, the sample size was calculated to be 172 with a relative precision of 20%, confidence level of 95%, power 80% and adding a non-response error of 20%.

Participants were approached in the waiting area of the outpatient departments of the hospitals and were briefed about the purpose and objectives of the study. After obtaining a written informed consent, data was collected from the willing participants by interviewing them personally using a questionnaire. Mothers with at least one child visiting the obstetric and pediatrics outpatient departments were included in the study using non-random (convenient) sampling technique.

This study was approved by the Institutional Ethics Committee of Kasturba Medical College, Mangalore (IEC KMC MLR 03-17).

### Data collection

Information was collected using a semi-structured questionnaire which had the following sections:

- *Section A:* Socio-demographic information of the participants (age, education, occupation, marital status, education and occupation of spouse, family income, number of family members, religion, place of residence, place of routine immunization, number of children, gender of the children);
- *Section B*: Core VH survey questions ( Larson *et al.*, 2015);
- *Section C*: Parental attitude towards childhood vaccination (PACV) ( Opel *et al.*, 2011). The PACV is a validated questionnaire which assesses the attitude of mothers towards vaccination under three domains: immunization behavior, beliefs about vaccine safety and efficacy and trust.

The questionnaire was translated to the local vernacular language, Kannada ( *Extended data* ( Rekha *et al.*, 2021)) and was pretested and content validated for the language. The pretesting was done on 30 mothers visiting the outreach clinics of the department of community medicine. Based on the responses, the questionnaire was modified for the language without changing the content. The content validation of the questionnaire was done by the subject expert. Socioeconomic status was evaluated using Modified B.G Prasad scale ( Singh, Sharma & Nagesh, 2017). Questionnaires were checked for completeness and only the completed questionnaires were considered for final analysis.

### Data management and analysis

The collected data was entered in and analyzed using IBM SPSS (Statistical Package for Social Sciences) Statistics for Windows Version 25.0. Armonk, NY: IBM Corp) and expressed using mean (Standard Deviation), and proportions. The calculated PACV scores ranged from 0-100: participants who scored between 0-49 were considered to have no VH, and those with scores from 50-100 were considered to be vaccine hesitant. The factors associated with VH was assessed using Chi-square test and P<0.05 was considered a statistically significant association.

## Results

A total of 172 mothers were assessed regarding VH. The mean age of the participants was 29.4 (±5.2) years, with majority (n=112, 65.1%) in the age group between 26-35 years. A higher proportion (n=61, 35.5%) of the participants had completed high school and were from middle socio-economic status. (n=30, 30%) Government health facilities were the place of routine immunization for 60.5% (n=104) of the participants. The socio-demographic characteristics of the study participants is depicted in Table 1.

**Table 1.** Socio-demographic characteristics of the study participants (N=172).

Variables	n (%)
**Age of mother (years)**	
≤25	40 (23.3)
26-35	112 (65.1)
>35	20 (11.6)
**Education of mother**	
Illiterate	13 (7.6)
Primary school	5 (2.9)
Middle school	50 (29.1)
High school	61 (35.5)
Post high school diploma	11 (6.4)
Graduate	25 (14.5)
Profession or Honors	7 (4.0)
**Occupation of mother**	
Unemployed	116 (67.4)
Unskilled worker	32 (18.6)
Semi-skilled worker	7 (4.1)
Skilled worker	3 (1.7)
Clerical/Shop-owner/Farmer	4 (2.3)
Professional	10 (5.8)
**Religion**	
Hindus	132 (76.7)
Muslims	34 (19.8)
Christian	6 (3.5)
**Socioeconomic status (B.G. Prasad scale) [N=100]**	
Upper class	19 (19.0)
Upper middle class	18 (18.0)
Middle class	30 (30.0)
Lower middle class	24 (24.0)
Lower class	9 (9.0)
**Place of residence**	
Urban	86 (50.0)
Rural	86 (50.0)
**Place of routine immunization**	
Government hospital	104 (60.5)
Private hospital	41 (23.8)
Anganwadi	27 (15.7)

Health care providers (n =79, 46%) were the major source of information regarding vaccines, followed by the child’s primary doctor (n= 52, 30.2%). Family members, television, newspaper magazine and internet comprised the rest of the sources.

Table 2 shows the findings of the baseline VH survey among the study participants. Only 7.6% (n=13) of the study participants had refused vaccination for their child at some point of them and the most common reason cited for vaccine refusal was that they thought it was not safe (n=6).

**Table 2.** Baseline vaccine hesitancy survey findings among the study participants (N=172).

Variable	n (%)
Belief that vaccines can protect children from serious diseases	169 (98.3)
Belief that most parents get their children vaccinated with all recommended vaccines	167 (97.1)
Reluctant/hesitant to get a vaccination for the child	19 (11.0)
Refused a vaccination for the child	13 (7.6)
**Reasons for vaccine refusal (N=13)** *	
Did not think the vaccine was safe	6 (46.2)
Distance of the immunization clinic	4 (30.8)
Did not know where to get reliable information	4 (30.8)
Someone else told me that the vaccine was not safe	4 (30.8)
Long waiting period in the immunization clinic	3 (23.1)
Heard or read negative media	3 (23.1)
Someone else told me that their child had a bad reaction	3 (23.1)
Fear of needles	3 (23.1)
Time needed to get to the clinic	2 (15.4)
Did not know where to get vaccinated	2 (15.4)
Not possible to leave other work	2 (15.4)
Did not think the vaccine was effective	2 (15.4)
Timings of the clinic	1 (7.7)
Had a bad experience or reaction with previous vaccination	1 (7.7)

* Multiple responses.

The perception of the participants regarding vaccine safety and efficacy, trust and their immunization behavior as assessed by PACV is shown in Table 3. A lower proportion of the participants (14%, n=24) opined that children get more vaccines than that are good for them. A higher proportion (80.8%, n=139) of the participants worried about their child having serious side effects from the vaccine. However, the majority (91.8%, n=158) of the participants trust the information provided to them regarding vaccines. A very small proportion (n=6, 3.5%) of the study participants had refused getting their child immunized and 5.8% (n=10) were hesitant about childhood vaccination.

**Table 3.** Perception of the study participants regarding vaccine safety and efficacy, trust and their immunization behavior as assessed by PACV (N=172).

Vaccine safety and efficacy			
	**Strongly agree/ Agree**	**Not sure**	**Strongly disagree/ Disagree**
Children get more shots than are good for them.	24 (14.0)	36 (20.9)	112 (65.1)
Many of the illness’s shots prevent are severe	133 (77.3)	28 (16.3)	11 (6.4)
Better for the child to develop immunity by getting sick than to get a shot	22 (12.8)	44 (25.6)	106 (61.6)
Better for children to get fewer vaccines at the same time	31 (18.0)	40 (23.3)	101 (58.7)
	**Not at all concerned/ Not too concerned**	**Not sure**	**Somewhat concerned/ Very concerned**
Concerned about the child having a serious side effect from a shot	130 (75.6)	9 (5.2)	33 (19.2)
Concerned that any one of the childhood vaccines may not be safe	139 (80.8)	11 (6.4)	22 (12.8)
Concerned that a shot might not prevent the disease?	143 (83.1)	21 (12.2)	8 (4.7)
**Trust**			
	**Strongly agree/ Agree**	**Not sure**	**Strongly disagree/ Disagree**
I trust the information I receive about shots	158 (91.8)	12 (7.0)	2 (1.2)
I can openly discuss my concerns about shots with my child’s doctor.	141 (82.0)	5 (2.9)	26 (15.1)
**Immunization behavior**			
	**Yes**	**No**	**Don’t know**
Delayed getting vaccine for reasons other than illness or allergy	14 (08.1)	157 (91.3)	1 (0.6)
Refused a vaccine for reasons other than illness or allergy	6 (3.5)	166 (96.5)	-
Follow the recommended schedule if the mother had another infant	149 (86.6)	19 (11.1)	4 (2.3)
**Overall hesitancy about childhood vaccination**	**Not at all hesitant/ Not too hesitant**	**Not sure**	**Somewhat hesitant / Very hesitant**
	153 (89.0)	9 (5.2)	10 (5.8)

The median PACV score among participants was 10.0 with a total of 166 parents (96.5%). Only 6 (3.5%) participants were found to be vaccine hesitant. Among the participants who were found to have no VH, a higher proportion were under the age of 30 years, urban residents, belonged to Hindu religion and utilized government facility for immunization. Among the vaccine hesitant mothers, majority of them had studied up to high school (83.3%) and were unemployed (66.7%). No statistical significance association was observed between VH and mothers educational and employment status (P>0.05) ( Table 4).

**Table 4.** Factors associated with vaccine hesitancy (N=172).

Factors	Vaccine hesitancy		Chi Square, P value
	Yes (N=6) n (%)	No (N=166) n (%)	
**Age of the mother (years)**			
≤30	4 (66.7)	104 (62.7)	0.040, P>0.841
>30	2 (33.3)	62 (37.3)	
**Educational status of the mother**			
Up to high school	5 (83.3)	124 (74.7)	0.230, P>0.532
Beyond high school	1 (16.7)	42 (25.3)	
**Employment status of the mother**			
Unemployed	4 (66.7)	112 (67.5)	0.002, P>0.637
Employed	2 (33.3)	54 (32.5)	
**Religion**			
Hindu	1 (16.7)	131 (78.9)	3.238, P<0.276
Muslim	4 (66.7)	30 (18.1)	
Christian	1 (16.7)	5 (3.0)	
**Place of residence**			
Urban	4 (66.7)	82 (49.4)	0.691, P>0.341
Rural	2 (33.3)	84 (50.6)	
**Place of immunization**			
Government facility	5 (83.3)	126 (75.9)	0.309, P>0.629
Private facility	1 (16.7)	40 (24.1)	

Socioeconomic status	N = 4	N = 96	
(N=100)	n (%)	n (%)	
Upper and upper middle class	3 (75.0)	34 (35.4)	2.581, P>0.142
Middle, lower middle and lowerclass	1 (25.0)	62 (64.6)	

## Discussion

The success of immunization programs relies on high vaccination coverage and vaccine uptake rates. Maintaining a high timely vaccination coverage has resulted in effective control of vaccine preventable diseases (VPDs), among those vaccinated, as well as the unvaccinated by virtue of herd immunity. However, recent outbreaks of VPDs like measles and diphtheria in both developed and developing countries, has undermined the progress made by immunization programs implemented all over the world. High vaccination coverage rates are misleading and do not reflect the number of under-vaccinated and unvaccinated section of the population ( Kumar *et al.*, 2016). The global vigilance on immunization programs in developed countries has attributed recent outbreaks of measles to VH ( Shukla, 2019). However, the trend of VH is universal and not just limited to developed countries. The analysis of WHO/ UNICEF Joint Reporting Form data for VH for the years 2015-2017 revealed that VH was reported by more than 90% of the WHO member countries ( Lane *et al.*, 2018).

In our study, a very low proportion (3.5%) of the participants were found to be vaccine hesitant. Though the immunization coverage rate of 77% of the study area is higher than the national average, it is still low. Being a health facility based study, the low VH in our study does not reflect VH in the general population. The lower VH among the participants could be due to high literacy rate, better immunization coverage and services, and creation of awareness by grass root level workers which has resulted in high vaccine uptake rates. With an educational index of 0.958 and an overall Human Development Index (HDI) of 0.830, Mangalore has always been one of the better performing regions in the State of Karnataka (Shodhganga. Profile of Dakshina Kannada, n.d.) There is huge variation in the estimates of VH reported in primary studies from various parts of the world. This variation may be attributed to the use of different questionnaires and cut-off scores to categorize VH among the population. Assessment of hesitancy for an individual vaccine or the entire immunization schedule, as well as the age group of target population may also result in VH estimates of varying proportions. The VH reported in studies from different parts of India ranged between 14.1% to 83% ( Krishnamoorthy *et al.*, 2019, Agarwal *et al.*, 2019, Narayanan, Jayaraman & Gopichandran, 2018, Dasgupta *et al.*, 2018). Similar variation in the prevalence of VH is also observed in studies from other parts of the world. The VH was as low as 1.1% in a study in Guatemala ( Domek *et al.*, 2018) while in a study in Nigeria it was 76% (Larsen et al, 2015b). Other studies have reported VH in the range of 11.6% to 66% ( Dubé *et al*, 2016, Lane *et al.*, 2018, Mohd Azizi, Kew, Moy, 2017, Giambi *et al.*, 2018, Ferrante *et al.*, 2019, Napolitano, D’Alessandro & Angelilo, 2018, Ray *et al.*, 2018, Paterson, Chantler & Larson, 2018). It is evident that irrespective of the measurement tool used, VH is widely prevalent across countries, having an impact on the coverage rates of immunization and vaccine uptake among the targeted population. Hence, it is imperative to first identify these vaccine hesitant groups, and strategically work towards restoring their trust and belief towards the health system in general and vaccination ( Marti *et al.*, 2017). Although by definition, it includes people who delay or refuse vaccination, people who chose to vaccinate in spite of having doubts about the same should also be identified and their concerns addressed. This will ensure a better immunization coverage in the future.

Identifying and overcoming the reasons for VH is one of the most important challenge faced by immunization and program managers. VH is usually limited to a subgroup of the population whose decision-making process is driven by their socio-cultural, religious, and political standing and cannot be overcome by strengthening the health system or increasing vaccination services alone. Studies have shown that there is not one particular reason for refusing or delaying vaccination. The reasons are diverse and can vary from one person to another in a vaccine hesitant population. Globally, the three most common reasons cited for VH during the year 2015, 2016 and 2017 were concerns related to vaccine safety (22%, 23%, 23%), lack of knowledge and awareness regarding immunization and its benefit (15%, 13%, 10%), and religious and cultural beliefs (10%, 9%, 12%) ( Marti *et al.*, 2017).

The reasons for VH varied across WHO regions and socio-economic status of the countries within these regions. In the European Region, South East Asian Region and Western Pacific Region, concerns about safety of vaccines and fear of side effects were predominant, while issues revolving around religion and cultural beliefs were common in the Americas. Inadequate knowledge or information regarding vaccines and their benefits as well as poor awareness were mainly reported from African Region and Eastern Mediterranean Region. Adverse events following immunization and safety of the vaccine were the most important driver for VH in upper-middle income countries, while inadequate knowledge and awareness regarding vaccines and immunization services were predominantly seen in lower-middle income countries. VH due to religious influences were seen across countries of all strata of income and in most of the WHO regions (The History of Vaccines. n.d.).

Apprehensions regarding the risks and benefit of vaccines were the most common reasons for VH in our study which was also reported by a study in Chennai. The other reasons for influencing VH cited in that study were lack of trust for the newer vaccines ( Narayanan, Jayaraman & Gopichandran, 2108). In a study in Siliguri, unwillingness and having no reliable information on the vaccine were the major reasons cited for VH ( Dasgupta *et al.*, 2018). Primary studies from different part of the world have also reported a heterogeneous group of reasons. The most frequently cited reason leading to VH in a study in England were the presence of porcine gelatin in the vaccine and issues related to vaccine effectiveness and side effects ( Lane *et al.*, 2018). Meanwhile, in a study in Guatemala, health system related factors like distance of the clinic, cost incurred to get to the clinic and timing of the clinic and waiting at the clinic were the predominant reasons for VH ( Domek *et al.*, 2018). A study conducted in Quebec found that fear of adverse effects and low perceived vulnerability of the child or severity of the disease were the most common reasons for VH ( Dubé *et al.*, 2016).

Religious influences for VH are the most challenging to address in any immunization program since they arise out of individual’s core belief and driven by their faith. Use of human tissues in creating a vaccine or the belief that the body should be healed naturally by God and not by chemicals are some of the points for refusing vaccines ( McKee & Bohannon, 2016). Unlike other reasons for VH, they do not arise out of lack of knowledge or awareness regarding immunization, but is a by-product of an individual’s or a group’s religious conviction thus making it difficult to change their attitude towards immunization ( McKee & Bohannon, 2016).

Issues related to vaccine safety and side effects is one of the most important reasons for refusal of vaccines by the parents. Parents are bombarded with information on vaccine safety and adverse effects, and opinions on vaccination through mass media and social media. Such information can be overwhelming at times, creating enough doubts to make an informed decision, thus leading them to ultimately refuse vaccination ( McKee & Bohannon, 2016).

Vaccine hesitancy among the cohort was not significantly associated with factors such as maternal age, employment, mother's educational status, religion, place of residence, place of immunization and socio-economic status (P>0.05). Mother’s age ( Krishnamoorthy *et al.*, 2019), lower educational status of mother ( Agarwal *et al.*, 2019, Dasgupta *et al.*, 2018) nuclear family ( Agarwal *et al.*, 2019, Dasgupta *et al.*, 2018), and past history of incomplete immunization ( Agarwal *et al.*, 2019) were found to be significantly associated with VH in other studies from India. Some of the factors associated with VH reported in studies from other parts of the world include younger parents ( Mohd Azizi, Kew & Moy, 2017), unemployed parents ( Mohd Azizi, Kew & Moy, 2017), higher educational status ( Ferrante *et al.*, 2019), contact with parents of children who had experienced serious side effects ( Giambi, 2018), not advised by a pediatrician to complete the full course of immunization ( Giambi, 2018), and lack of trust on the pediatrician ( Napolitano, 2018).

### Limitations

This being a facility-based study, our findings cannot be extrapolated to the general population.

## Conclusion

Our study highlights the presence of very low prevalence of VH in Mangalore compared to similar studies from India and other parts of the world. The limited number of participants who had refused vaccination, refused due to concerns related to vaccine safety.

### Recommendations

Hesitancy to vaccines follows the iceberg phenomenon, with the tip of the iceberg representing the population who refuse vaccination, while the submerged proportion representing the vaccine hesitant population. Identifying these vaccine hesitant subgroups is important for the success of any immunization program. Population-based studies covering a larger sample and multicentric studies would throw more evidence towards the presence of VH as a reason for a lower vaccination. Such studies also give an insight into the various reasons for VH existing in different population groups. 

## Data availability

### Underlying data

Open Science Framework: Vaccine Hesitancy among mothers of Under-5 children in Coastal South India - A facility-based study, https://doi.org/10.17605/OSF.IO/A3XJU ( Rekha *et al.*, 2021).

### Extended data

Open Science Framework: Vaccine Hesitancy among mothers of Under-5 children in Coastal South India - A facility-based study, https://doi.org/10.17605/OSF.IO/A3XJU ( Rekha *et al.*, 2021).

This project contains the following extended data:

- Questionnaire as asked in the local language.

Data are available under the terms of the Creative Commons Zero “No rights reserved” data waiver (CC0 1.0 Public domain dedication).
