# Supplementary material for: Vaccine hesitancy among mothers of under-five children in Coastal South India: a facility-based cross-sectional study
Source: F1000Res. 2021 Sep 16;10:186. Originally published 2021 Mar 8. [Version 3] doi: 10.12688/f1000research.28293.3 (PMC8477350; doi:10.12688/f1000research.28293.3)
Supplement: Peer review history

# Peer review: Reviewer response for version 3

**DOI:** 10.5256/f1000research.77220.r94488

**Published:** 2021-09-27

**Authors:** Aida Kalok

**Affiliations:** 1 Department of Obstetrics and Gynaecology, Faculty of Medicine, National University of Malaysia, Universiti Kebangsaan Malaysia Medical Centre, Selangor, Malaysia

Thank you for adding the p values in table 4. You should however state the value as 'p equals( p = )', instead of using > or <.

 Thanks.

Is the work clearly and accurately presented and does it cite the current literature?

Yes

If applicable, is the statistical analysis and its interpretation appropriate?

Partly

Are all the source data underlying the results available to ensure full reproducibility?

Yes

Is the study design appropriate and is the work technically sound?

Yes

Are the conclusions drawn adequately supported by the results?

Yes

Are sufficient details of methods and analysis provided to allow replication by others?

Yes

Reviewer Expertise:

Maternal health, General Obstetrics.

I confirm that I have read this submission and believe that I have an appropriate level of expertise to confirm that it is of an acceptable scientific standard.

# Peer review: Reviewer response for version 2

**DOI:** 10.5256/f1000research.58578.r91978

**Published:** 2021-08-24

**Authors:** Aida Kalok

**Affiliations:** 1 Department of Obstetrics and Gynaecology, Faculty of Medicine, National University of Malaysia, Universiti Kebangsaan Malaysia Medical Centre, Selangor, Malaysia

Thank you for the revised version.

 A couple more comments prior to my approval of this manuscript:

1. Please include the exact p-value of each chi-square test in Table 4.
2. This statement is quite confusing: "In our study, mothers aged less than 30 years, educated up to high school and unemployed, residing in an urban area, belonging to Muslim religion, and availing immunization services from a government facility were found to be vaccine hesitant, though these factors were not significant (P>0.05)".
  Please consider rephrasing it to "Vaccine hesitancy among the cohort was not significantly associated with factors such as maternal age, employment, religion, urban residence, etc."

Is the work clearly and accurately presented and does it cite the current literature?

Yes

If applicable, is the statistical analysis and its interpretation appropriate?

Partly

Are all the source data underlying the results available to ensure full reproducibility?

Yes

Is the study design appropriate and is the work technically sound?

Yes

Are the conclusions drawn adequately supported by the results?

Yes

Are sufficient details of methods and analysis provided to allow replication by others?

Yes

Reviewer Expertise:

Maternal health, General Obstetrics.

I confirm that I have read this submission and believe that I have an appropriate level of expertise to confirm that it is of an acceptable scientific standard, however I have significant reservations, as outlined above.

# Response

**Published:** 2021-08-30

**Authors:** Nithin Kumar

**Affiliations:** Kasturba Medical College Hospital, India

Thank you for taking time and reviewing our article. We have made the changes in the manuscript and uploaded the revised version. Kindly find the point-by-point clarification/reply to your comments.

- Please include the exact p-value of each chi-square test in Table 4.           

         
**Thank you. The exact P value is incorporated in table 4 as per your suggestion.**

  

- This statement is quite confusing: "In our study, mothers aged less than 30 years, educated up to high school and unemployed, residing in an urban area, belonging to Muslim religion, and availing immunization services from a government facility were found to be vaccine hesitant, though these factors were not significant (P>0.05)".

          Please consider rephrasing it to "Vaccine hesitancy among the cohort was not significantly associated with factors such as maternal age, employment, religion, urban residence, etc."     

     

**Thank you for your suggestion. We have rephrased the statement as per your suggestion.**

# Peer review: Reviewer response for version 1

**DOI:** 10.5256/f1000research.31291.r82849

**Published:** 2021-05-04

**Authors:** Aida Kalok

**Affiliations:** 1 Department of Obstetrics and Gynaecology, Faculty of Medicine, National University of Malaysia, Universiti Kebangsaan Malaysia Medical Centre, Selangor, Malaysia

- The introduction is quite lengthy.
- Sample size - please state the number of participants required based on the sample size calculation. Did this study reach its required sample size?
- What was the study hypothesis?
- Data collection: Did the participants complete the questionnaire themselves? What about the illiterate mothers?
- Data analysis: Was there any association between VH and maternal employment/level of education/socio-economic status? Please include this in the results/discussion.
- Discussion:
  - Please discuss why with such a low VH rate, the proportion of immunised children in Mangalore is still around 77%.
  - The study was conducted in a health facility; women who sought/received health care were most likely to demonstrate a lower level of vaccine hesitancy.

Is the work clearly and accurately presented and does it cite the current literature?

Yes

If applicable, is the statistical analysis and its interpretation appropriate?

Partly

Are all the source data underlying the results available to ensure full reproducibility?

Yes

Is the study design appropriate and is the work technically sound?

Yes

Are the conclusions drawn adequately supported by the results?

Yes

Are sufficient details of methods and analysis provided to allow replication by others?

Yes

Reviewer Expertise:

Maternal health, General Obstetrics.

I confirm that I have read this submission and believe that I have an appropriate level of expertise to confirm that it is of an acceptable scientific standard, however I have significant reservations, as outlined above.

# Response

**Published:** 2021-07-09

**Authors:** Nithin Kumar

**Affiliations:** Kasturba Medical College Hospital, India

*We thank you for taking out your valuable time to review our article. We have incorporated all the changes and corrections suggested by you in the manuscript. Kindly find the response letter below.*

**The introduction is quite lengthy.**

 Thank you for the suggestion. We have made the introduction concise and specific, in line with the objectives of the study.

**Sample size - please state the number of participants required based on the sample size calculation. Did this study reach its required sample size?**

 Based on the sample size calculation, a total of 172 participants were required to be included in the study. We were able to achieve the sample size. The same has been added to the Methods section in the manuscript.

**What was the study hypothesis?**

 Even though the study area has a good health system and a literacy rate that is higher than the national figures, the immunization coverage has consistently remained below 90 per cent. The study hypothesis was “Vaccine hesitancy is present in the study area”.

**Data collection: Did the participants complete the questionnaire themselves? What about illiterate mothers?**

 All the participants were interviewed while they were waiting in the respective OPDs. The same has been included in the methods section of the manuscript.

**Data analysis: Was there any association between VH and maternal employment/level of education/socio-economic status? Please include this in the results/discussion.**

 Crosstabulation between the presence of vaccine hesitancy and educational status, employment status and socioeconomic status is incorporated in the results and discussion section.  However, no statistically significant association was observed between VH and maternal employment, level of education and socio-economic status.

**Discussion:  Please discuss why with such a low VH rate, the proportion of immunized children in Mangalore is still around 77%.**

 Being a health facility-based study, our finding of low VH among the study participants does not reflect the status of VH in Mangalore. Population-based studies covering a larger sample / multicentric studies would throw more evidence towards the presence of VH as a reason for a lower vaccination coverage in the study area. Also, since the study was conducted in a health facility; the women who sought/received

  health care were most likely to demonstrate a better health seeking behavior which is also reflected in  a lower vaccine hesitancy among the participants.

**The study was conducted in a health facility; women who sought/received health care were most likely to demonstrate a lower level of vaccine hesitancy.   **                   

 Yes. we agree with the observation.

# Peer review: Reviewer response for version 1

**DOI:** 10.5256/f1000research.31291.r81112

**Published:** 2021-03-22

**Authors:** Giriyanna Gowda

**Affiliations:** 1 Department of Community Medicine, Kempegowda Institute of Medical Sciences, Bangalore, Karnataka, India

Comments:

1. The introduction is too lengthy; please make it specific and concise and based on the needs of the study.
2. In the abstract, it is mentioned that the prevalence of vaccine hesitance is 3.4 % and also that 7.6 % had refused vaccination for their child. Please clarify this.
3. Recommendations are not based on results. Kindly specify it.

Is the work clearly and accurately presented and does it cite the current literature?

Yes

If applicable, is the statistical analysis and its interpretation appropriate?

Yes

Are all the source data underlying the results available to ensure full reproducibility?

Yes

Is the study design appropriate and is the work technically sound?

Yes

Are the conclusions drawn adequately supported by the results?

Yes

Are sufficient details of methods and analysis provided to allow replication by others?

Yes

Reviewer Expertise:

epidemiology, clinical trials

I confirm that I have read this submission and believe that I have an appropriate level of expertise to confirm that it is of an acceptable scientific standard.

# Response

**Published:** 2021-07-09

**Authors:** Nithin Kumar

**Affiliations:** Kasturba Medical College Hospital, India

*We thank you for taking out your valuable time to review our article. We have incorporated all the changes and corrections suggested by you in the manuscript. Kindly find the response letter below.*

**Response to the Comments:**

**1.The introduction is too lengthy; please make it specific and concise and based on the needs of the study.**

 Thank you for the suggestion. We have made the introduction concise and specific, in line with the objectives of the study.

**2. In the abstract, it is mentioned that the prevalence of vaccine hesitance is 3.4 % and that 7.6 % had refused vaccination for their child. Please clarify this.**One of the questions in the Core vaccine hesitancy Survey tool directed towards the mothers was “Have you ever refused a vaccination for your child? Yes/No”.  Only 7.6% of the study participants had refused vaccination for their child at some point of time.

 The prevalence of vaccine hesitancy was calculated based on the scores obtained from the Parental Attitude Towards Childhood Vaccination Questionnaire (PACV). Participants who scored between 50 -100 were vaccine hesitant. Only 3.4% of the study participants were vaccine hesitant.  We have clarified it in abstract.

**3. Recommendations are not based on results. Kindly specify it.**

 Recommendations have been made specific to the conclusions drawn from the study.
